# Bacterial tail anchors can target to the mitochondrial outer membrane

**DOI:** 10.1186/s13062-017-0187-0

**Published:** 2017-07-24

**Authors:** Güleycan Lutfullahoğlu-Bal, Abdurrahman Keskin, Ayşe Bengisu Seferoğlu, Cory D. Dunn

**Affiliations:** 1 Department of Molecular Biology and Genetics, Koç University, 34450 Sarıyer, İstanbul, Turkey; 2 Institute of Biotechnology, Helsinki Institute of Life Science, University of Helsinki, P.O. Box 56, 00014 Helsinki, Finland; 3 Present Address: Department of Biological Sciences, Columbia University, New York, NY 10027 USA

**Keywords:** Protein targeting, Membrane insertion, Eukaryogenesis, Organelle biogenesis, Endosymbiosis

## Abstract

**Background:**

During the generation and evolution of the eukaryotic cell, a proteobacterial endosymbiont was re-fashioned into the mitochondrion, an organelle that appears to have been present in the ancestor of all present-day eukaryotes. Mitochondria harbor proteomes derived from coding information located both inside and outside the organelle, and the rate-limiting step toward the formation of eukaryotic cells may have been development of an import apparatus allowing protein entry to mitochondria. Currently, a widely conserved translocon allows proteins to pass from the cytosol into mitochondria, but how proteins encoded outside of mitochondria were first directed to these organelles at the dawn of eukaryogenesis is not clear. Because several proteins targeted by a carboxyl-terminal tail anchor (TA) appear to have the ability to insert spontaneously into the mitochondrial outer membrane (OM), it is possible that self-inserting, tail-anchored polypeptides obtained from bacteria might have formed the first gate allowing proteins to access mitochondria from the cytosol.

**Results:**

Here, we tested whether bacterial TAs are capable of targeting to mitochondria. In a survey of proteins encoded by the proteobacterium *Escherichia coli*, predicted TA sequences were directed to specific subcellular locations within the yeast *Saccharomyces cerevisiae*. Importantly, TAs obtained from DUF883 family members ElaB and YqjD were abundantly localized to and inserted at the mitochondrial OM.

**Conclusions:**

Our results support the notion that eukaryotic cells are able to utilize membrane-targeting signals present in bacterial proteins obtained by lateral gene transfer, and our findings make plausible a model in which mitochondrial protein translocation was first driven by tail-anchored proteins.

**Reviewers:**

This article was reviewed by Michael Ryan and Thomas Simmen.

**Electronic supplementary material:**

The online version of this article (doi:10.1186/s13062-017-0187-0) contains supplementary material, which is available to authorized users.

## Background

During the incorporation of an α-proteobacterial endosymbiont within the eukaryotic cell, genes transferred to the (proto)nucleus were re-targeted to mitochondria, allowing these organelles to remain the location of crucial cellular processes [1–3]. In addition, other polypeptides that evolved within the eukaryotic lineage or that were acquired through lateral gene transfer from other organisms were directed to mitochondria [4–6]. Across eukaryotes, the β-barrel Tom40 protein forms a pore by which proteins pass through the OM [7–9]. However, the Tom40 polypeptide seems to require already existing TOM complexes for mitochondrial insertion [10, 11], giving rise to a “chicken or the egg” dilemma when considering how the TOM complex may have evolved.

Several narratives might be proposed for how mitochondria first evolved the ability to transport proteins from the cytosol. In one scenario, an early translocation pore that was self-inserting at the mitochondrial surface might have allowed mitochondria to begin to import proteins, permitting the subsequent evolution of the translocon found in eukaryotes today [12]. Current evidence suggests that the self-insertion of tail-anchored proteins at the mitochondrial OM is possible [13–15], and some tail-anchored pro-apoptotic proteins appear to have the ability to generate membrane pores at mitochondria [16, 17], making tenable such a scenario for the evolution of mitochondrial protein import. At the inception of mitochondria, such tail-anchored proteins would likely have been derived from prokaryotes, particularly if mitochondria were required for the generation of the stereotypical compartmentalized structure of eukaryotes.

We focused our attention upon a single aspect of this hypothesis: can TAs obtained from bacterial proteins be inserted into the mitochondrial OM when expressed within a eukaryotic cell? Indeed, our results demonstrate insertion and function at the mitochondrial OM for predicted TAs encoded by the proteobacterium *E. coli*, and we describe the relevance of our findings to the concept of lateral gene transfer during eukaryogenesis.

## Results

### Bacterial tail anchors can localize to mitochondria

**Fig. 1:**
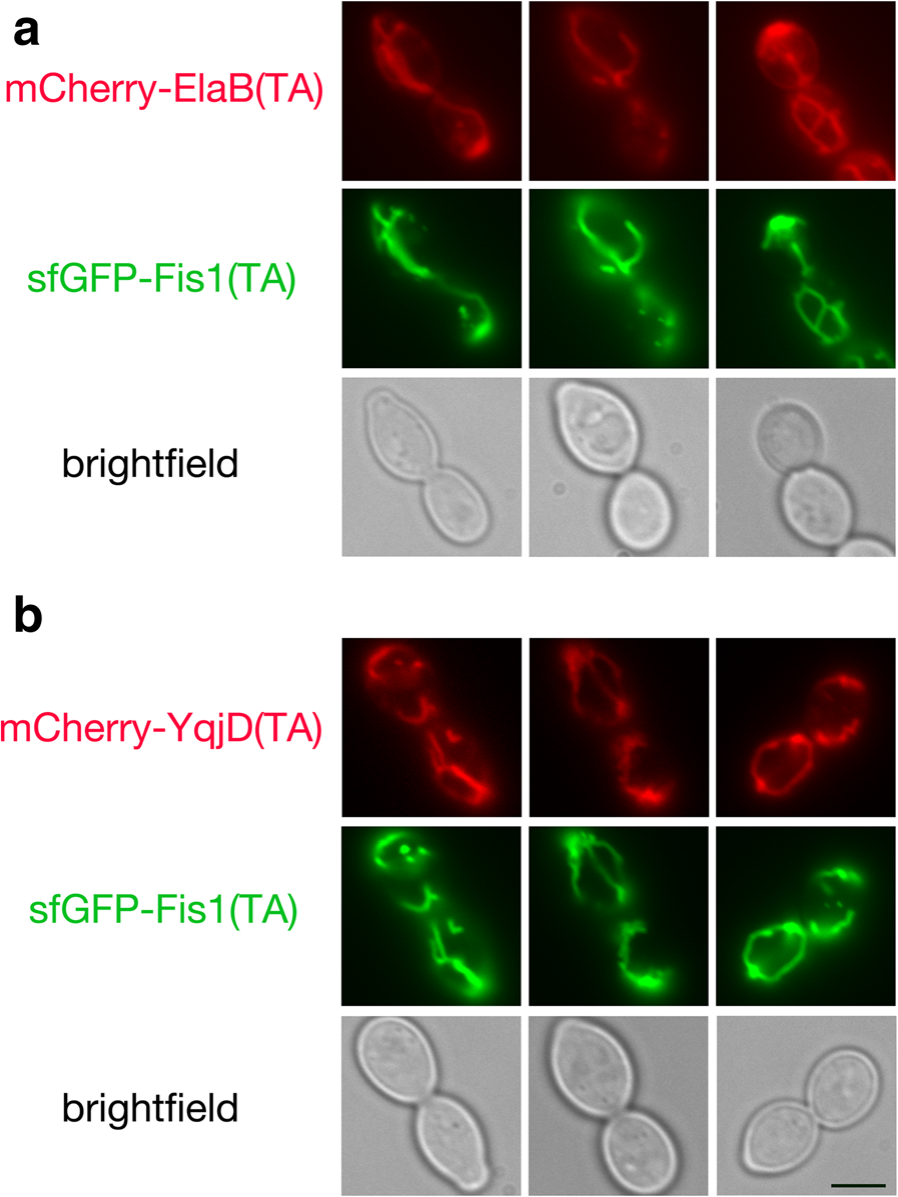
The predicted ElaB and YqjD TAs localize to mitochondria. Strain BY4741, harboring plasmid b294 (sfGFP-Fis1p), was mated to strain BY4742 carrying mCherry-ElaB(TA)-expressing plasmid b275 (**a**) or mCherry-YqjD(TA)-expressing plasmid b279 (**b**). The resulting diploids were visualized by fluorescence microscopy. Scale bar, 5 μm

To test whether predicted bacterial TAs might have the capacity to be inserted at the mitochondrial OM, we identified 12 *E. coli* proteins predicted to harbor a solitary α-helical transmembrane (TM) domain at the polypeptide carboxyl-terminus (Additional file 1: Figure S1), then fused mCherry to the amino-terminus of these TAs and examined their location in *S. cerevisiae* cells by fluorescence microscopy. mCherry-ElaB(TA) (Fig. 1a) and mCherry-YqjD(TA) (Fig. 1b) were readily detectable at mitochondria, as reported by co-localization with superfolder GFP (sfGFP) [18] fused to the TA of the *S. cerevisiae* Fis1 polypeptide, a protein playing a role in yeast mitochondrial division. A small fraction of mCherry-ElaB(TA) and mCherry-YqjD(TA) could also be detected at the endoplasmic reticulum (ER) (Additional file 2: Figure S2). ElaB and YqjD are members of the DUF883 family of proteins. Little is known about the function of DUF883 family members, but YqjD may recruit ribosomes to the *E. coli* plasma membrane during stationary phase [19].

Due to dual localization of mCherry-ElaB(TA) and mCherry-YqjD(TA), we investigated whether the targeting of these proteins might be influenced by ER-mitochondria encounter structures (ERMES). However, mCherry-ElaB(TA) and mCherry-YqjD(TA) were not limited to ERMES, as defined by Mdm34p-containing puncta [20] (Additional file 3: Figure S3), and disruption of ERMES by deletion of Mdm34p did not affect distribution of these fusion proteins to the swollen mitochondria resulting from Mdm34p removal [21, 22] or in their limited localization to ER (Additional file 4: Figure S4).

Although negligible fluorescent signal was detectable by microscopy or flow cytometry (C. Dunn, unpublished results), mCherry-TcdA(TA) could also be visualized at mitochondria (Additional file 5: Figure S5A). TcdA (also called CsdL) catalyzes the modification of *E. coli* tRNAs [23]. Other predicted TAs derived from the *E. coli* proteins Flk, YgiM, RfaJ, DjlB, FdnH, NrfF, and YmiA appeared to allow at least partial localization of mCherry to various locations associated with the endomembrane system (Additional file 6: Figure S6). However, no convincing localization to mitochondria was apparent after fusing any of these TAs to mCherry. The mCherry-YhdV(TA) fusion protein appeared to be distributed throughout cytosol and nucleus, indicating failure to target efficiently to any membrane. mCherry-YgaM(TA) was not detectable, suggesting its degradation.

### Bacterial tail anchors can insert into membranes in a eukaryotic cell

**Fig. 2:**
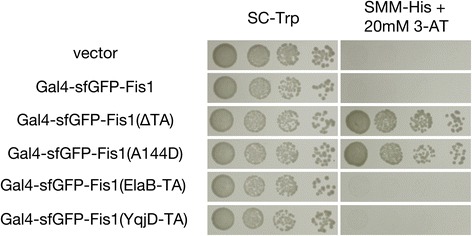
A proliferation-based assay suggests that the ElaB and YqjD TAs are membrane inserted. Strain MaV203, containing a Gal4-driven *HIS3* gene, was transformed with plasmids expressing Gal4-sfGFP-Fis1p (b100), a variant lacking the Fis1p TA (b101), a mutant containing the A144D charge substitution in its TA (b180), or the Gal4-sfGFP-Fis1p construct with the Fis1p TA replaced with that of either ElaB (b313) or YqjD (b314). MaV203 was also transformed with empty vector pKS1. Transformants were cultured in SC-Trp medium, then, following serial dilution, spotted to SC-Trp or SMM-His +20 mM 3-AT and incubated for 2 d

Previously, we developed an assay in which membrane insertion of proteins might be examined by a proliferation-based assay [24]. In brief, the Gal4 transcription factor is linked to a protein of interest that is thought to be inserted at a membrane outside of the nucleus. Failure of this fusion protein to insert at its target membrane can allow the Gal4-linked fusion protein to access the nucleus and activate Gal4-responsive promoters that drive proliferation under selective conditions. As previously demonstrated [24], while a membrane-sequestered Gal4-sfGFP-Fis1 fusion protein did not lead to a proliferation defect on non-selective medium (SC-Trp), cells carrying this construct could not survive on medium requiring activation of a Gal4p-driven *HIS3* gene (SMM-His +20 mM 3-AT) (Fig. 2). Deletion of the Fis1p TA, or the presence of a A144D charge substitution within the Fis1p TA, led to a failure of membrane insertion at mitochondria, translocation to the nucleus, and Gal4-dependent proliferation on selective medium. When the TA of Fis1p, a domain whose sole purpose is to allow this protein’s insertion at the mitochondrial OM [24, 25], was replaced with the TA of either ElaB or YqjD, cells were unable to proliferate on medium selective for histidine synthesis, consistent with ElaB and YqjD TA insertion at the mitochondrial OM.

### Bacterial tail anchors can function at the mitochondrial outer membrane

**Fig. 3:**
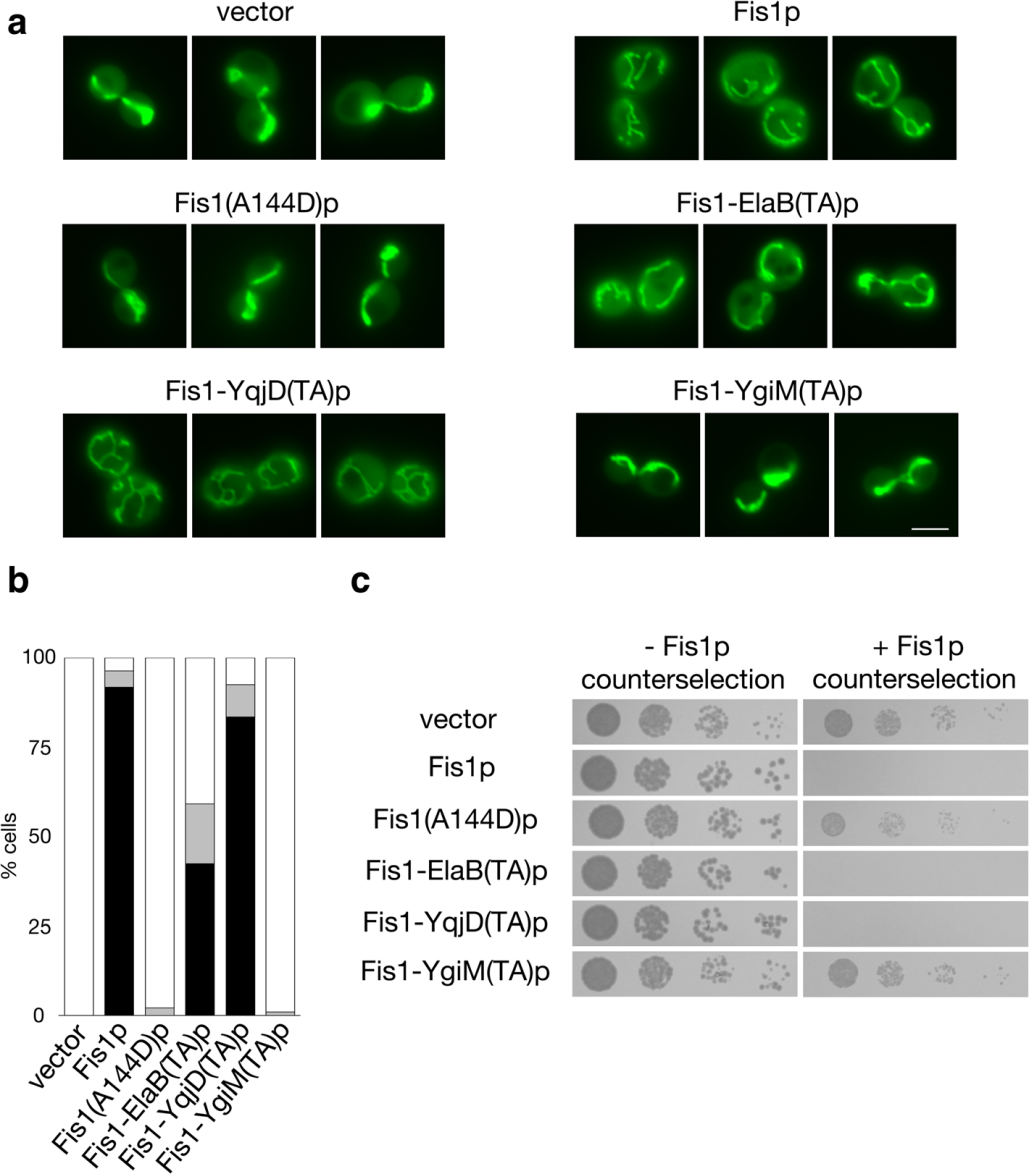
Mitochondria-localized bacterial TAs can functionally replace the TA of Fis1p. **a** The ElaB and YqjD TAs can replace the Fis1p TA in promotion of normal mitochondrial morphology. *fis1∆* strain CDD741, expressing mitochondria-targeted GFP from plasmid pHS12, was transformed with empty vector pRS313 or plasmids expressing wild-type Fis1p (b239), Fis1(A144D)p (b244), or Fis1p with its own TA replaced by that of ElaB (b317), YqjD (b318), or YgiM (b316). Cells were examined by fluorescence microscopy. Scale bar, 5 μm. **b** Quantification of mitochondrial morphology of the transformants from (**a**) was performed blind to genotype. White bar represents cells with fully networked mitochondria, grey bar represents cells with mitochondria not fully networked, but networked to a greater extent than wild-type cells, and black bar represents cells with normal mitochondrial morphology. Quantification was repeated three times (*n* > 200 per genotype), and a representative experiment is shown. **c** Genetic assessment of Fis1p variant functionality. Strain CDD688 was transformed with the plasmids used in (**a**) and proliferation was assessed without selection against Fis1p activity (YPALac medium for 2 d) or following counter-selection for cells carrying functional Fis1p (SLac-His + CHX medium for 4 d)

As these findings suggested that the ElaB and YqjD TAs may be competent for mitochondrial insertion, we tested whether these TAs can functionally replace the membrane-bound TA of Fis1p, thereby allowing Fis1p to promote mitochondrial division. Because Fis1p is required for mitochondrial fission in *S. cerevisiae*, mutants lacking this protein manifest a highly interconnected network of mitochondria due to unbalanced mitochondrial fusion [26–28]. As expected, expression of wild-type Fis1p restored normal mitochondrial distribution in this genetic background, while Fis1p prevented from mitochondrial insertion by a A144D substitution within the Fis1p TA [24] could not restore normal mitochondrial morphology (Fig. 3a and b). Strikingly, replacement of the Fis1p TA with the ElaB or the YqjD TA within the context of full length Fis1p polypeptide could successfully promote mitochondrial division and restore normal mitochondrial shape and distribution. A control TA obtained from the *E. coli* YgiM protein, which is not trafficked to mitochondria, could not support Fis1p activity. In addition, a Fis1-TcdA(TA) protein could not functionally replace the Fis1p TA in this microcopy-based assay (Additional file 5: Figure S5B), suggesting insufficient expression, poor mitochondrial insertion, or meager functionality.

We then sought further evidence for functional insertion of the ElaB and YqjD TAs at the mitochondrial OM using an assay based upon cell proliferation [24]. Continued mitochondrial division after removal of a mitochondrial fusogen, such as Fzo1p, can lead to mitochondrial fragmentation, loss of functional mitochondrial DNA (mtDNA), and a corresponding abrogation of respiratory competence [29–32]. This phenotype can be used as a basis to test Fis1p functionality [24]. As previously reported, expression of wild-type Fis1p in a *fzo1∆ fis1∆* genetic background led to an inability to proliferate on non-fermentable medium, while expression of the poorly inserted Fis1(A144D) variant did not prompt mtDNA loss (Fig. 3c). The ElaB and YqjD TAs fused to the cytosolic domain of Fis1p allowed sufficient fission activity to prompt mitochondrial genome loss from the same genetic background, indicating successful ElaB TA and YqjD TA insertion at the mitochondrial OM. Even the Fis1-TcdA(TA) protein provoked mtDNA loss in *fzo1∆ fis1∆* cells (Additional file 5: Figure S5C), suggesting some minimal level of OM insertion, while the YgiM TA again appeared unable to recruit Fis1p to mitochondria (Fig. 3c). Together, our results demonstrate insertion of the bacterial ElaB and YqjD TAs at the mitochondrial surface of a eukaryotic cell.

## Discussion

Our findings, in which several predicted TAs obtained from *E. coli* can target to and function at the mitochondrial OM of *S. cerevisiae*, make plausible a scenario in which tail-anchored bacterial proteins contributed to the formation of the earliest mitochondrial translocon. The structural characteristics of the TAs of ElaB and YqjD, a helical TM domain rich in glycines followed by a positively charged patch ending in di-arginine (Additional file 1: Figure S1), are evocative of the Fis1p TA, suggesting a similar, potentially spontaneous mechanism for insertion at mitochondria, although unassisted insertion of the ElaB and YqjD TAs at the mitochondrial surface has yet to be demonstrated. Notably, several conserved members of the current TOM complex are also tail-anchored [33], raising the possibility that at least some of these proteins could be “hold-overs” from an early, self-inserting mitochondrial translocon, although we note that these subunits cannot currently self-insert at mitochondria.

Could the DUF883 family of proteins have contributed to an ancestral mitochondrial OM translocon? While YqjD has been reported to recruit ribosomes to the *E. coli* inner membrane during stationary phase [19], a role in line with promotion of co-translational protein import into mitochondria [34, 35], the DUF883 family is not readily identified among eukaryotic genomes. One might expect, however, that once a more proficient TOM complex centered around the Tom40 pore evolved, a previous translocon would have been lost, or even selected against if it were to interfere with more rapid protein import through an improved OM translocation machinery. Moreover, an inordinate focus on DUF883 family members when seeking components of the earliest mitochondrial translocon may not be warranted in any case, since the structural characteristics likely required for TA insertion at mitochondria might be easily generated from random open reading frame fragments containing a transmembrane domain. Analogously, random peptides from bacteria are readily able to act as amino-terminal mitochondrial targeting sequences [36–38]. If TAs are easily evolved and might recruit other functional domains to the mitochondrial surface, then identifying orthologs of initial tail-anchored translocon components from existing prokaryotic sequences might be difficult, since an untold number of TAs might be predicted among putative open-reading frames. Supporting the idea that mitochondrial TAs might be generated from sequences not actually functioning in membrane targeting within their native bacterial environment, we demonstrated limited mitochondrial targeting and partial functionality of the computationally predicted TcdA TA in yeast, even though TcdA is unlikely to be membrane-inserted in *E. coli* [39].

If conversion of endosymbiont to mitochondrion were the rare and essential event required for generation of eukaryotes, and if insertion of bacteria-derived, tail-anchored proteins at the OM to form an ancestral translocon were necessary for this conversion, then the question of how hospitable an environment the early mitochondrial OM might have been for bacteria-derived TAs comes to the fore [12]. Indeed, the membrane into which tail-anchored proteins are inserted can be at least partially determined by their lipid environment [13], and lipids utilized by many characterized archaea are fundamentally different in structure from bacterial and eukaryotic lipids [40]. However, recent evidence indicates that archaeal clades potentially related to the last eukaryotic common ancestor might have been characterized by membranes more similar to those of bacteria than of those membranes more typically found in archaea [41]. This finding raises the possibility that the proto-eukaryote’s specific cohort of lipids was crucial to the ability to form complexes of bacteria-derived tail-anchored proteins at the mitochondrial OM that might have allowed full integration of mitochondria within the ancestral eukaryote.

Finally, we have not examined in detail the trafficking of *E. coli* TAs that appeared to localize to the endomembrane system during our initial survey. However, the diverse organellar locations to which these TAs were localized supports previous data indicating that eukaryotes may derive organelle targeting information from newly acquired prokaryotic proteins or protein fragments, perhaps even from amino acid sequences previously unselected for targeting proficiency [36–38, 42, 43]. Lateral gene transfer promotes the evolution of novel functions in prokaryotes [44] and was certainly present in the form of endosymbiotic gene transfer during early eukaryogenesis. Indeed, proficiency in making use of cryptic or explicit targeting information in order to direct newly acquired, nucleus-encoded proteins to the distinct subcellular locations where they might be best utilized might have provided a significant selective advantage to the early eukaryote. Such a scenario may be particularly relevant if some amount of cellular compartmentalization already existed in a pre-eukaryotic host cell before conversion of pre-mitochondrial endosymbiont to organelle [45, 46].

## Conclusions

We have demonstrated that TAs from bacteria can localize to and insert within the mitochondrial OM. Our results make plausible the suggestion that tail-anchored proteins acquired by bacteria could have formed an initial translocon at the mitochondrial outer membrane, and our findings indicate that certain membrane-bound proteins acquired by horizontal gene transfer could have easily found their way to diverse locations within eukaryotic cells at which they might provide a selective advantage. Further efforts will be necessary to determine whether self-inserting proteins or peptides may have generated the initial mitochondrial translocon.

## Methods

### Yeast strains, plasmids, and culture conditions

Culture conditions are as described in [24], and all experiments have been carried out at 30 °C. Strains, plasmids, and oligonucleotides used in this study are found in Additional file 7.

### Selection of *E. coli* tail anchors subject to investigation

FASTA sequences from the *E. coli* proteome were retrieved from UniProt [47] and analyzed using the TMHMM 2.0 server [48]. Polypeptides with a single predicted TM domain, harboring 15 or less amino acids carboxyl-terminal to the TM domain, and containing more than 30 amino acids amino-terminal to the TM domain were selected for further analysis.

### Microscopy

Microscopy was performed on logarithmic phase cultures as in [24], with exposure times determined automatically. mCherry fusions are driven by the *ADH1* promoter and universally contain Fis1p amino acids 119-128 (not necessary or sufficient for mitochondrial targeting) linking mCherry to each TA. The brightness of all images of mCherry expression was adjusted in Adobe Photoshop CS5 (Adobe, San Jose, California) to an equivalent extent. Microscopic assessment of Fis1p variant functionality was performed as described in [24], and scoring of mitochondrial morphology was performed blind to genotype.

### Proliferation-based assessment of Fis1p insertion and functionality

Genetic tests of Fis1p insertion and functionality were performed as in [24].

## Reviewers’ comments

### Reviewer 1: Michael T. Ryan, Monash University

#### General comments

In this manuscript, Bal and colleagues address elements of the endosymbiotic hypothesis ­ notably whether tail­anchored proteins from bacteria can directly insert into mitochondria and therefore have the potential to have formed the first components of the mitochondrial protein import machinery. While the translocation channel is a beta barrel and likely came from bacteria, the evolutionary origin of single transmembrane receptor proteins is not well established. Here the authors definitively show that a subset of tail anchored bacterial proteins can indeed target mitochondria when expressed in yeast cells. The work is very clear and the manuscript is exceedingly well written. I think this is an interesting and important piece of work that merits publication. I have no issues to report.

#### Specific comments

I have no issues with this manuscript ­ it is extremely well written and well presented.

Authors’ Response: *We thank Dr. Ryan for his encouraging comments regarding our manuscript.*

### Reviewer 2: Thomas Simmen, University of Alberta

#### General comments

Bal et al. present a thought­provoking study that investigates an intriguing question: can today’s bacterial tail­anchored proteins provide mitochondrial targeting information within a eukaryotic cell. The article warrants interest by a larger audience, but for the moment, some shortcomings must be addressed to make the manuscript more succinct and convincing. More specifically, some of the data are of rather subpar quality and there are some questions about the mtDNA depletion assay, which would lead some readers to dismiss the study. Given the originality of the topic, this should provide some solid insight into this attractive hypothesis.

#### Specific comments

This is a thought­provoking study that investigates an intriguing question: can today’s bacterial tail­anchored proteins provide mitochondrial targeting information within a eukaryotic cell. The article should generate interest by a larger audience, but for the moment, some shortcomings must be addressed to make the manuscript more succinct and convincing. The proposed improvements aim to make this a stronger paper.

Specific Point 1. The microscopic technique used at the moment is unclear; the images appear a little blurred to draw the rather precise conclusions that the authors propose. For the localization studies that the paper advertises, confocal or deconvolution microscopy must be used.

Authors’ Response 1: *It is typical to detect some out-of-focus light from spherical yeast cells when using epifluorescence light microscopy. Moreover, our experience with standard confocal microscopy of yeast is that image quality is generally not improved, due to reduced signal intensity and diminished ability to visualize mitochondrial morphology throughout the cell. To summarize, we feel that co-localization of mCherry-ElaB(TA) and mCherry-YqjD(TA) with mitochondria is well documented here, with little to be gained by use of alternative microscopy approaches*.

*Incidentally, we noted upon upload of our files for this revision that the resolution is much reduced in the PDF generated for review. We can directly provide the 600dpi files upon request before publication, if the reviewer wishes.*

Specific Point 2. The authors state that some constructs localize to the ER, but that is not obvious. It might be interesting to pursue this question, since some mitochondrial TA proteins might transit to mitochondria on ER­mitochondria contact sites (see also below). Due to the potential relevance of this question, some colocalization should be done.

Authors’ Response 2: *Only a minor fraction of mCherry-ElaB(TA) and mCherry-YqjD(TA) constructs localize to ER. However, these proteins are quite clearly detectable at this subcellular location, as defined by use of Sec63p-GFP as an ER marker, in several of the representative images collected.*

*We agree that it is worthwhile to examine potential localization of these tail anchors with respect to ER-mitochondria contact sides. Toward this goal, we compared the localization of mCherry-ElaB(TA) and mCherry-YqjD(TA) to that of an ER-mitochondria encounter structure (ERMES) component, Mdm34p, fused to superfolder GFP. However, we found no evidence of concentration of the ElaB and YqjD TAs at ERMES. These new results are found in* Additional file 3: Figure S3.

*In addition, we disrupted ERMES by removal of Mdm34p, but we detected no change to the localization of mCherry-ElaB(TA) or mCherry-YqjD(TA): the majority of each protein was targeted to mitochondria and a minority of each protein was found at the ER. These data are provided in* Additional file 4: Figure S4.

Specific Point 3. mCherry­ TcdA(TA) is claimed to partially localize to mitochondria. I cannot see this with the current image quality. This should be improved and potentially complemented with fractionation studies.

Authors’ Response 3: *As described in the text, the signal of mCherry-TcdA(TA) is quite weak, suggesting that the protein may be unstable. Much of the mCherry-TcdA(TA) that can be detected appears generally distributed throughout the cytosol of the cell. This might be expected for a sequence that, contrary to our computational prediction, does not actually function as a TA in bacteria *[39]. *However, some small fraction of the mCherry-TcdA(TA) construct is evidently directed to mitochondria, as indicated by co-localization with sfGFP-Fis1(TA). Most importantly, mtDNA loss occurring specifically when Fis1-TcdA(TA)p is expressed in cells lacking both Fzo1p and wild-type Fis1p* (Additional file 5: Figure S5C) *demonstrates by genetic means that the TcdA TA can be inserted at the mitochondrial outer membrane.*

Specific Point 4. On page 5 at the beginning of the bottom chapter, the authors claim that their results suggest that ElaB and YqiD mediate insertion into mitochondrial membranes, but at this point of the text, they have only presented evidence for membrane, not mitochondrial insertion.

Authors’ Response 4: *We thank the reviewer for this correction, and we have changed the relevant text from, “As these findings indicated...” to “As these findings suggested...”*

Specific Point 5. On Fig. 3a, i do not see any evidence that ElaB TA rescues targeting of Fis1 and, hence, mitochondrial fission.

Authors’ Response 5: *We maintain that our images show an overt rescue of mitochondrial morphology for cells expressing Fis1-ElaB(TA)p when compared to cells expressing non-functional Fis1(A144D)p or Fis1-YgiM(TA)p, or not expressing any Fis1p variant at all (vector control). Indeed, mitochondrial morphology was also quantified blind to genotype (*Fig. 3b *), further validating our conclusion that Fis1-ElaB(TA)p is functional.*

Specific Point 6. Since TcdA also leads to loss of mtDNA in S3C, but does not show mitochondrial targeting, the authors should provide an assay, which can detect also small amounts of mitochondrial targeting, ideally fractionation.

Authors’ Response 6: *Some mCherry-TcdA(TA) is localized to mitochondria, as described above (Specific Point 3) and seen in (what is now)* Additional file 5: Figure S5A.

*Moreover, our genetic assay argues for some minimal amount of mitochondrial insertion, since Fis1-TcdA(TA)p can provoke mtDNA loss in cells deleted of both Fis1p and Fzo1p. We expect that our genetic assay is a more sensitive readout of Fis1p function when compared to a microscopy-based assay. If sufficient fission takes place at any time during the generation of a cell pedigree, mtDNA is permanently lost. In contrast, a microscopy-based assay may not easily capture instances at which Fis1p construct activity is sufficiently high to promote rescue of mitochondrial morphology that would be readily discernable by fluorescence microscopy. To summarize, our localization of mCherry-TcdA(TA) to mitochondria, combined with the results of our genetic assay, demonstrate that a small population of proteins terminating in a predicted TcdA TA can insert into the mitochondrial outer membrane.*

*We would like to thank Dr. Simmen for his helpful comments regarding our manuscript.*

## Additional files

Additional file 1: Figure S1. A list of predicted TAs examined in this study. The UniProt accession number and names of selected proteins are provided, along with the sequences of the predicted TAs. The single predicted TM domain is denoted by a purple line. Charged amino acids are also indicated. For purposes of sequence comparison, the relevant portion of the *S. cerevisiae* Fis1p TA is also shown. (TIFF 526 kb)

Additional file 2: Figure S2. The predicted ElaB and YqjD TAs can also be visualized at the endoplasmic reticulum. Cells harboring (A) plasmid b275 [mCherry-ElaB(TA)] or (B) plasmid b279 [mCherry-YqjD(TA)] were analyzed as in Fig. 1, except BY4741 was transformed with plasmid pJK59, expressing Sec63p-GFP, before mating. In addition, cells expressing mCherry-Fis1(TA) from plasmid b109 are shown for comparison (C). Scale bar, 5 μm. (TIFF 5526 kb)

Additional file 3: Figure S3. The predicted ElaB and YqjD TAs are not specifically localized to ERMES. Strain CDD1210, expressing Mdm34p-sfGFP, was transformed with (A) plasmid b275 [mCherry-ElaB(TA)] or (B) plasmid b279 [mCherry-YqjD(TA)] and visualized by fluorescence microscopy. Scale bar, 5 μm. (TIFF 3859 kb)

Additional file 4: Figure S4. Disruption of ERMES does not affect trafficking of ElaB and YqjD TAs to mitochondria and ER. *mdm34∆* strain CDD1209 was transformed with plasmid b275 [mCherry-ElaB(TA)] (A and B) or plasmid b279 [mCherry-YqjD(TA)] (C and D). The mitochondrial matrix was labelled using pHS1 [Cox4p(1-21)-GFP] (A and C), and the ER membrane was labelled using pJK59 (Sec63p-GFP) (B and D). Cells were visualized by fluorescence microscopy. Scale bar, 5 μm. (TIFF 6631 kb)

Additional file 5: Figure S5. The predicted TcdA TA allows minimal localization to, and function at, the mitochondrial outer membrane. (A) The predicted TcdA TA can be visualized at mitochondria. Strain BY4741, harboring plasmid b294 (sfGFP-Fis1p), was mated to strain BY4742 carrying mCherry-TcdA(TA)-expressing plasmid b281 and the resulting diploids were imaged by fluorscence microscopy. Scale bar, 5 μm. (B) Fis1p with its own TA replaced by the predicted TcdA TA cannot provide detectable Fis1p activity as assessed by visualizing mitochondrial morphology. *fis1∆* strain CDD741, expressing mitochondria-targeted GFP from plasmid pHS12, was transformed with empty vector pRS313 or plasmids expressing wild-type Fis1p (b239), Fis1(A144D)p (b244), or Fis1-TcdA(TA)p (b319) and mitochondrial morphology was examined. (C) Fis1-TcdA(TA)p can allow mitochondrial division. Strain CDD688 was transformed with the plasmids used in (B) or a plasmid expressing Fis1-YgiM(TA)p (b316) and examined as in Fig. 3c, except that culture on medium counter-selective for Fis1p activity was carried out for 5 d. (TIFF 5312 kb)

Additional file 6: Figure S6. Not all predicted *E. coli* TAs are localized to mitochondria in *S. cerevisiae*. Strain CDD961 was transformed with plasmids expressing (A) mCherry-Flk(TA) (b273), (B) mCherry-YhdV(TA) (b277), (C) mCherry-RfaJ(RA) (b278), (D) mCherry-DjlB(TA) (b280), (E) mCherry-FdnH(TA) (b331), (F) mCherry-NrfF(TA) (b332), or (G) mCherry-YmiA(TA) (b333) and examined by fluorescence microscopy. (H) Strain BY4741, carrying plasmid b311 expressing sfGFP fused to the enhanced PTS1 sequence [49], was mated to strain BY4742, containing the mCherry-YgiM(TA)-expressing plasmid b274, and the resulting diploids were imaged. (TIFF 9892 kb)

Additional file 7: Strains, plasmids, and oligonucleotides used during this study [24, 32, 50–56]. (XLS 49 kb)

## Abbreviations

ER: Endoplasmic reticulum
ERMES: ER-mitochondria encounter structure
mtDNA: Mitochondrial DNA
OM: Outer membrane
sfGFP: Superfolder GFP
TA: Tail anchor
TM: Transmembrane
