# Supplementary figures and images for: Bacterial tail anchors can target to the mitochondrial outer membrane
Source: Biol Direct. 2017 Jul 24;12:16. doi: 10.1186/s13062-017-0187-0 (PMC5525287; doi:10.1186/s13062-017-0187-0)

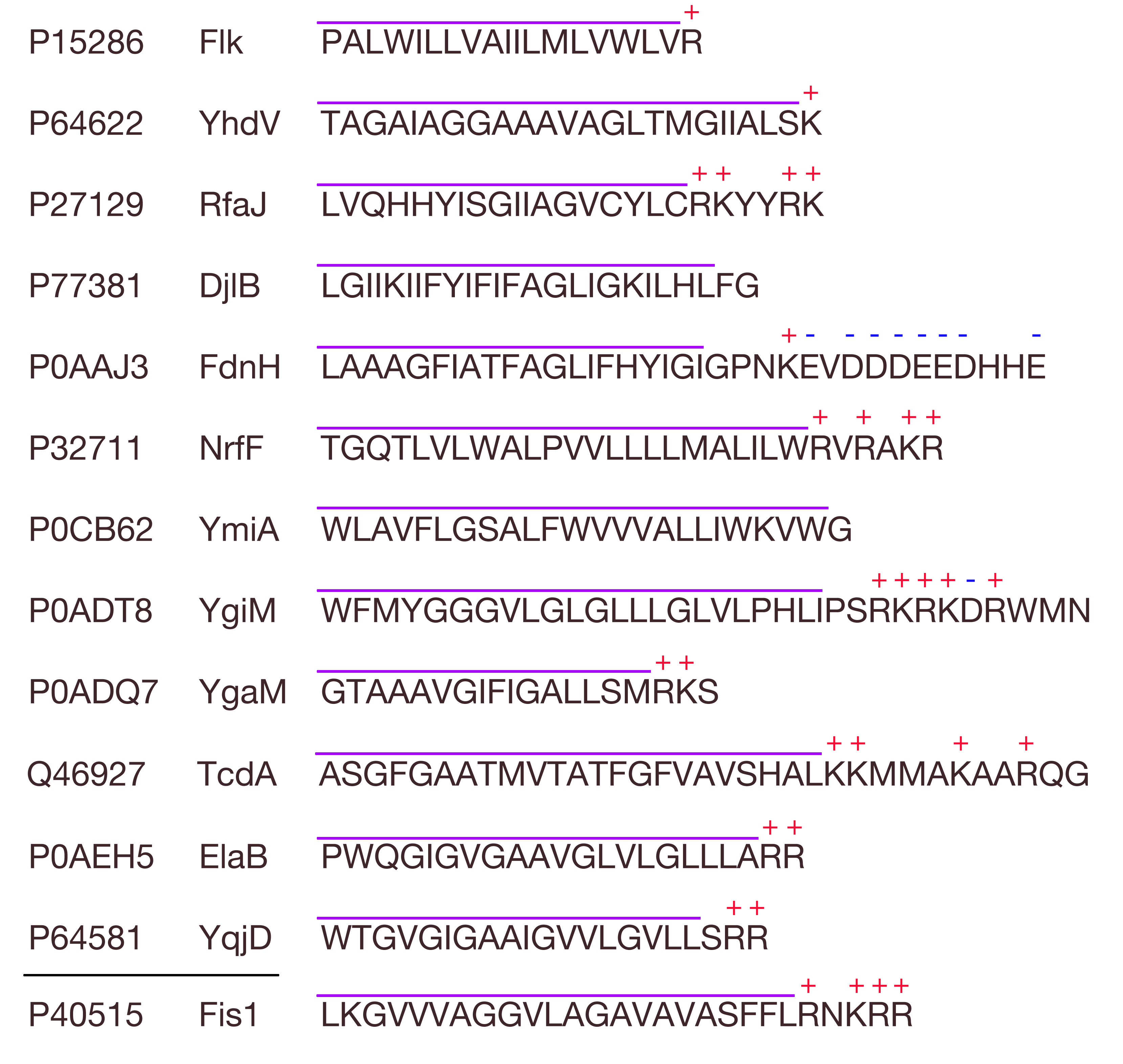

Supplement: Supplementary file 1 — A list of predicted TAs examined in this study. The UniProt accession number and names of selected proteins are provided, along with the sequences of the predicted TAs. The single predicted TM domain is denoted by a purple line. Charged amino acids are also indicated. For purposes of sequence comparison, the relevant portion of the S. cerevisiae Fis1p TA is also shown. (TIFF 526 kb) [file 13062_2017_187_MOESM1_ESM.tif]

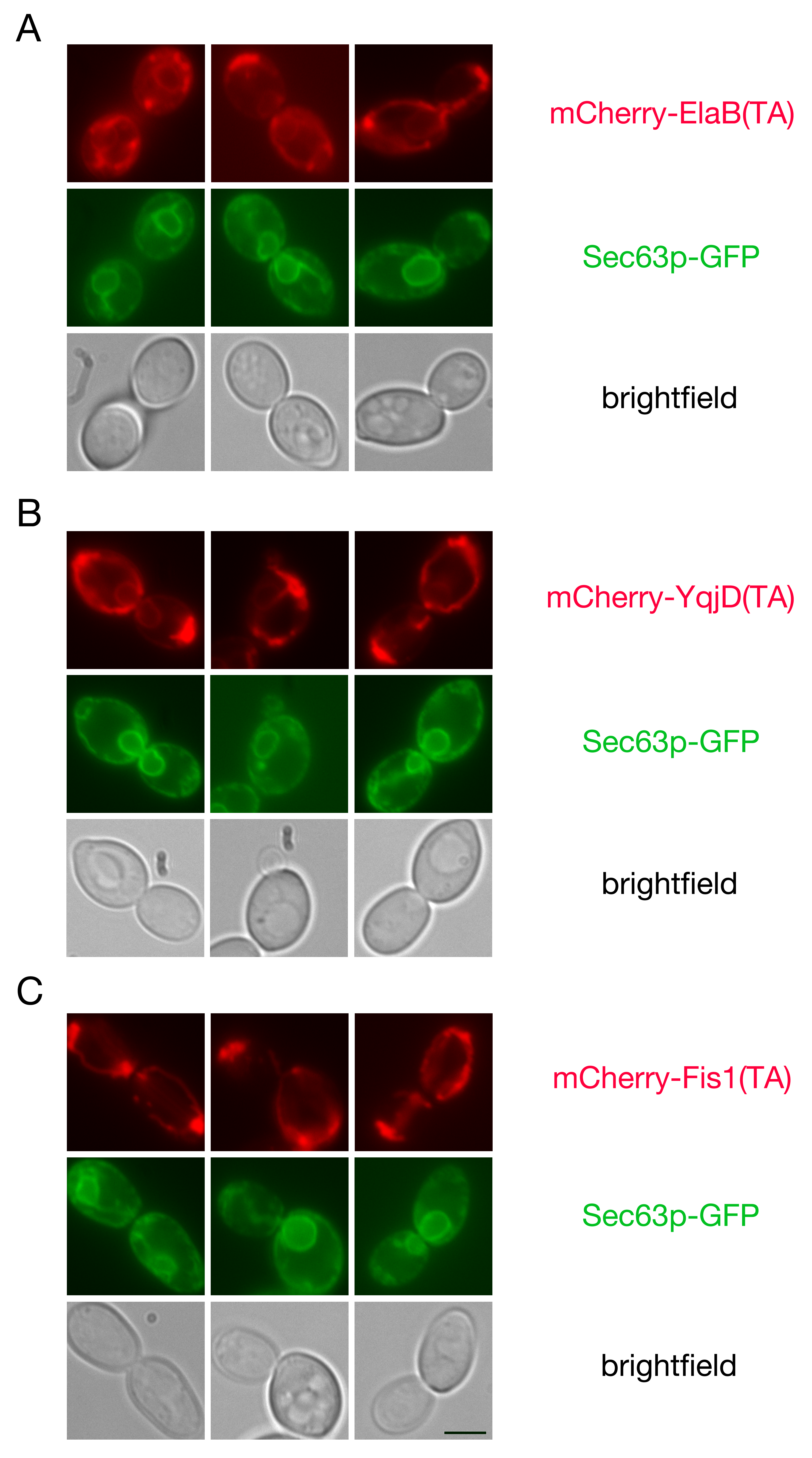

Supplement: Supplementary file 2 — The predicted ElaB and YqjD TAs can also be visualized at the endoplasmic reticulum. Cells harboring (A) plasmid b275 [mCherry-ElaB(TA)] or (B) plasmid b279 [mCherry-YqjD(TA)] were analyzed as in Fig. 1, except BY4741 was transformed with plasmid pJK59, expressing Sec63p-GFP, before mating. In addition, cells expressing mCherry-Fis1(TA) from plasmid b109 are shown for comparison (C). Scale bar, 5 μm. (TIFF 5526 kb) [file 13062_2017_187_MOESM2_ESM.tif]

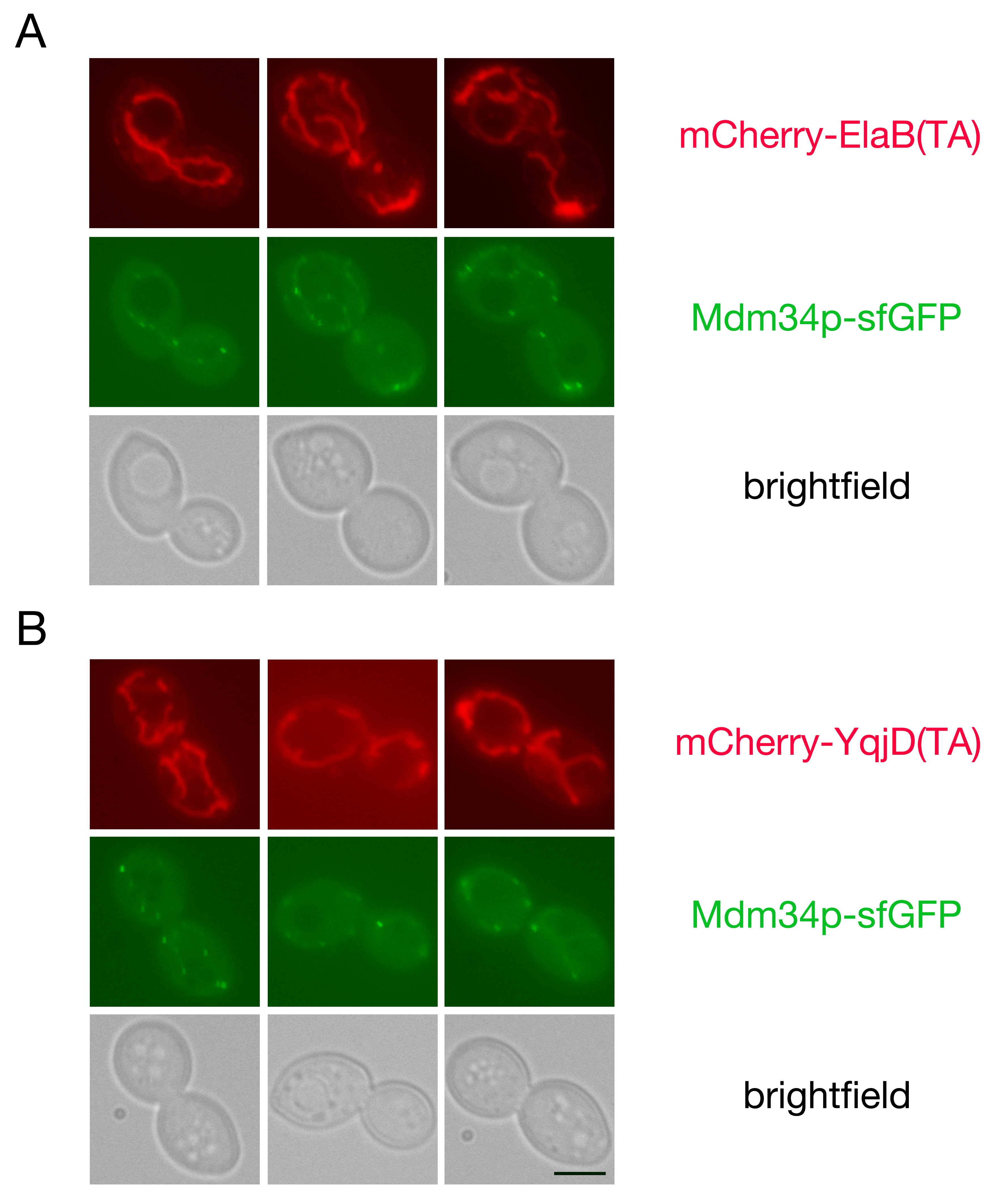

Supplement: Supplementary file 3 — The predicted ElaB and YqjD TAs are not specifically localized to ERMES. Strain CDD1210, expressing Mdm34p-sfGFP, was transformed with (A) plasmid b275 [mCherry-ElaB(TA)] or (B) plasmid b279 [mCherry-YqjD(TA)] and visualized by fluorescence microscopy. Scale bar, 5 μm. (TIFF 3859 kb) [file 13062_2017_187_MOESM3_ESM.tif]

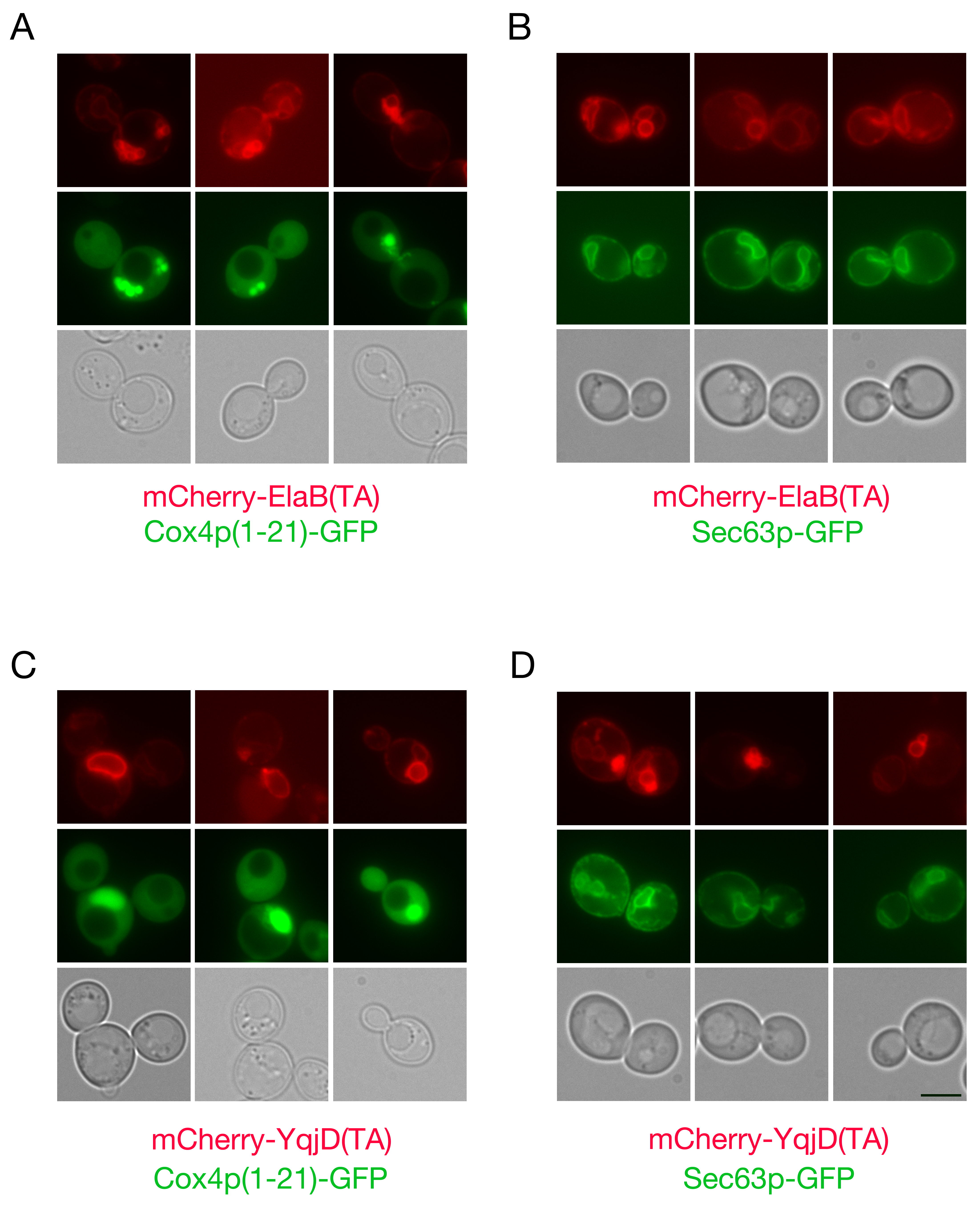

Supplement: Supplementary file 4 — Disruption of ERMES does not affect trafficking of ElaB and YqjD TAs to mitochondria and ER. mdm34∆ strain CDD1209 was transformed with plasmid b275 [mCherry-ElaB(TA)] (A and B) or plasmid b279 [mCherry-YqjD(TA)] (C and D). The mitochondrial matrix was labelled using pHS1 [Cox4p(1-21)-GFP] (A and C), and the ER membrane was labelled using pJK59 (Sec63p-GFP) (B and D). Cells were visualized by fluorescence microscopy. Scale bar, 5 μm. (TIFF 6631 kb) [file 13062_2017_187_MOESM4_ESM.tif]

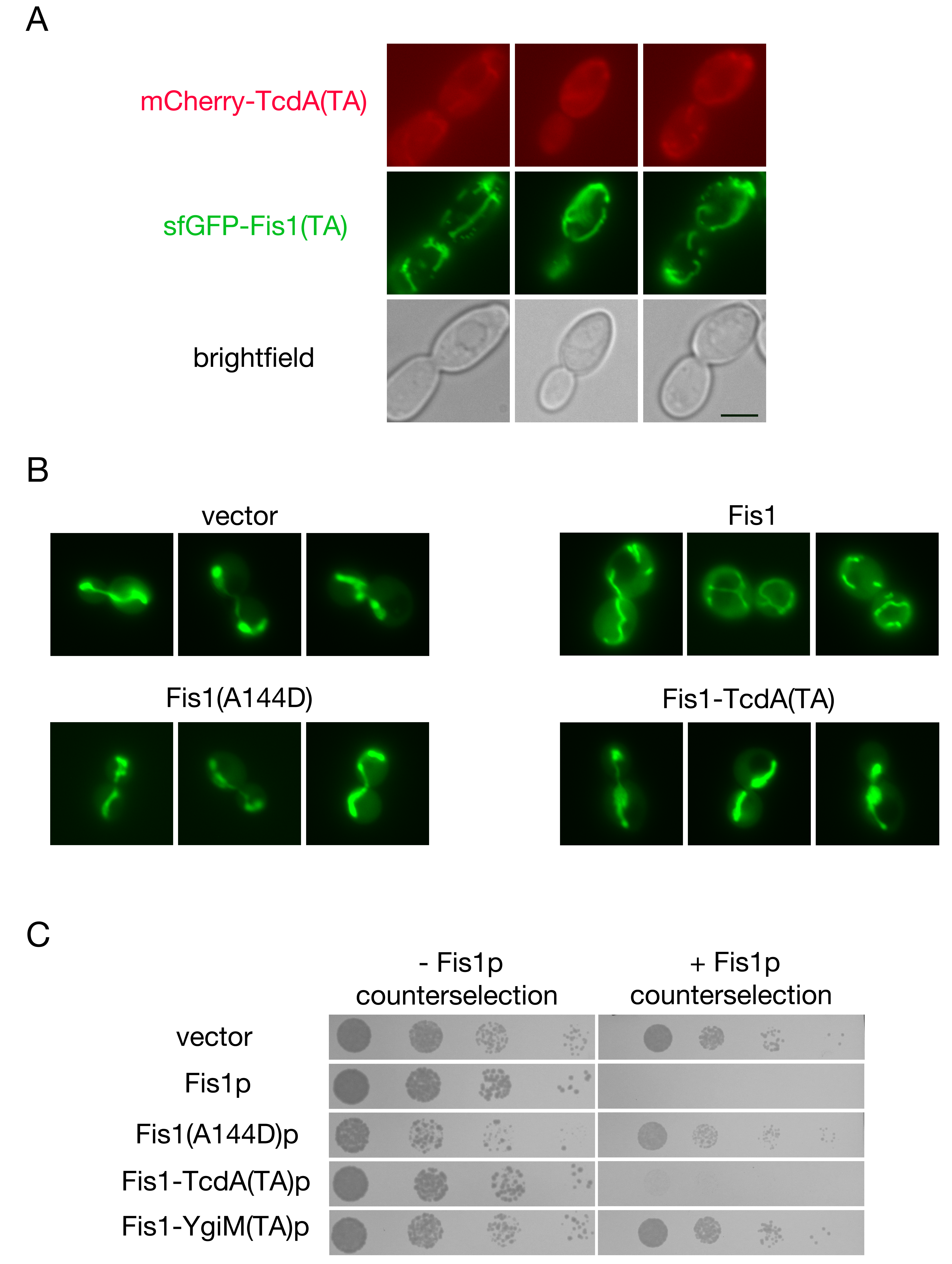

Supplement: Supplementary file 5 — The predicted TcdA TA allows minimal localization to, and function at, the mitochondrial outer membrane. (A) The predicted TcdA TA can be visualized at mitochondria. Strain BY4741, harboring plasmid b294 (sfGFP-Fis1p), was mated to strain BY4742 carrying mCherry-TcdA(TA)-expressing plasmid b281 and the resulting diploids were imaged by fluorscence microscopy. Scale bar, 5 μm. (B) Fis1p with its own TA replaced by the predicted TcdA TA cannot provide detectable Fis1p activity as assessed by visualizing mitochondrial morphology. fis1∆ strain CDD741, expressing mitochondria-targeted GFP from plasmid pHS12, was transformed with empty vector pRS313 or plasmids expressing wild-type Fis1p (b239), Fis1(A144D)p (b244), or Fis1-TcdA(TA)p (b319) and mitochondrial morphology was examined. (C) Fis1-TcdA(TA)p can allow mitochondrial division. Strain CDD688 was transformed with the plasmids used in (B) or a plasmid expressing Fis1-YgiM(TA)p (b316) and examined as in Fig. 3c, except that culture on medium counter-selective for Fis1p activity was carried out for 5 d. (TIFF 5312 kb) [file 13062_2017_187_MOESM5_ESM.tif]

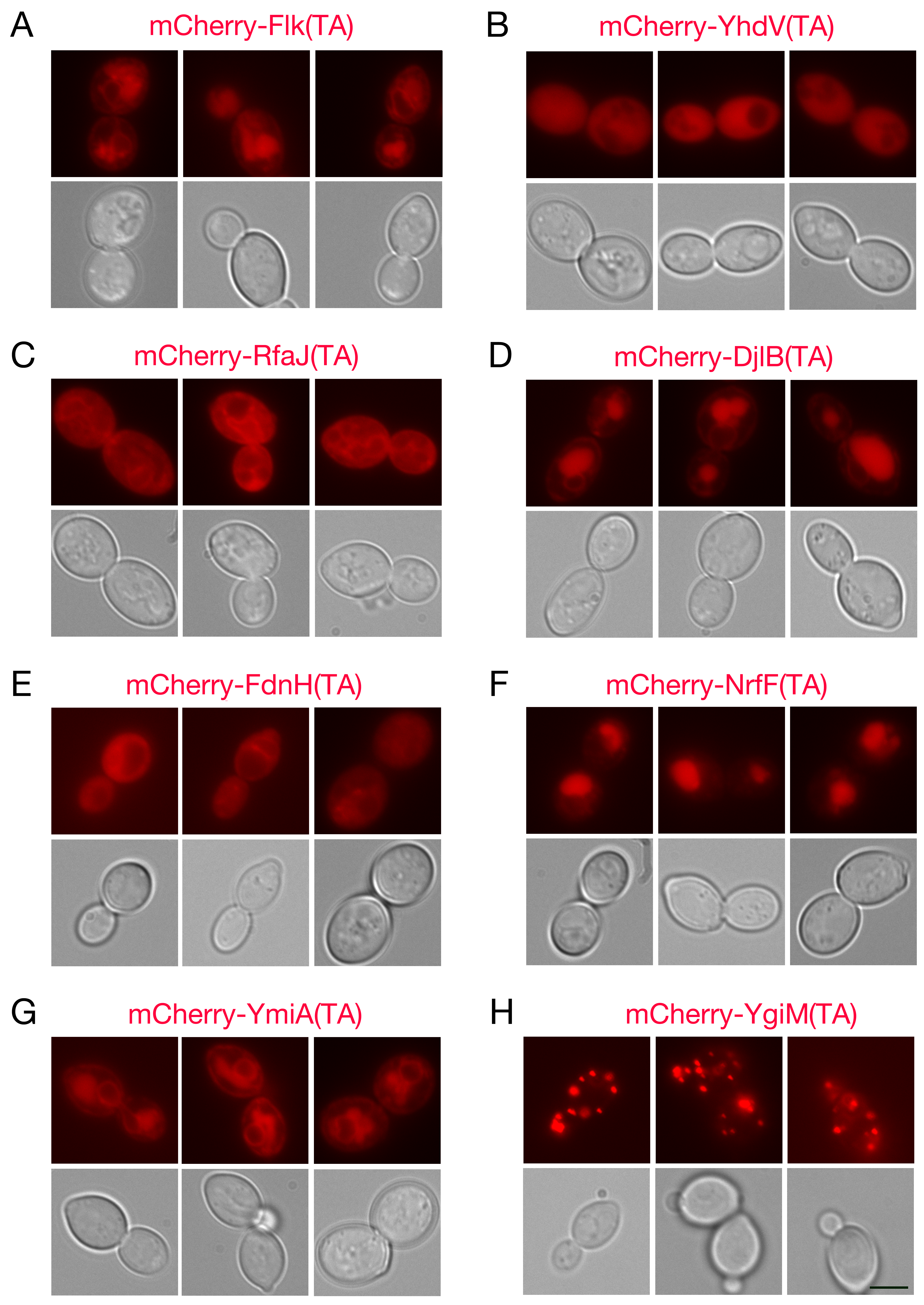

Supplement: Supplementary file 6 — Not all predicted E. coli TAs are localized to mitochondria in S. cerevisiae. Strain CDD961 was transformed with plasmids expressing (A) mCherry-Flk(TA) (b273), (B) mCherry-YhdV(TA) (b277), (C) mCherry-RfaJ(RA) (b278), (D) mCherry-DjlB(TA) (b280), (E) mCherry-FdnH(TA) (b331), (F) mCherry-NrfF(TA) (b332), or (G) mCherry-YmiA(TA) (b333) and examined by fluorescence microscopy. (H) Strain BY4741, carrying plasmid b311 expressing sfGFP fused to the enhanced PTS1 sequence [49], was mated to strain BY4742, containing the mCherry-YgiM(TA)-expressing plasmid b274, and the resulting diploids were imaged. (TIFF 9892 kb) [file 13062_2017_187_MOESM6_ESM.tif]
